# From global action against malaria to local issues: state of the art and perspectives of web platforms dealing with malaria information

**DOI:** 10.1186/s12936-018-2270-0

**Published:** 2018-03-21

**Authors:** Dominique Briand, Emmanuel Roux, Jean Christophe Desconnets, Carmen Gervet, Christovam Barcellos

**Affiliations:** 1 FIOCRUZ, LIS Laboratory, Avenida Brasil, 4365, Pavilhão Haity Moussatché, Rio de Janeiro, Brazil; 2 IRD, UMR ESPACE-DEV, Maison de la télédétection, 500 rue Jean François Breton, 34090 Montpelllier, France; 3 Université de Montpellier, UMR ESPACE-DEV, Maison de la télédétection, 500 rue Jean François Breton, 34090 Montpellier, France

**Keywords:** Web technologies, Information systems, Metadata, Web of linked data, Multidisciplinarity, Interoperability

## Abstract

**Background:**

Since prehistory to present times and despite a rough combat against it, malaria remains a concern for human beings. While evolutions of science and technology through times allowed for some infectious diseases eradication in the 20th century, malaria resists.

**Objectives:**

This review aims at assessing how Internet and web technologies are used in fighting malaria. Precisely, how do malaria fighting actors profit from these developments, how do they deal with ensuing phenomena, such as the increase of data volume, and did these technologies bring new opportunities for fighting malaria?

**Methods:**

Eleven web platforms linked to spatio-temporal malaria information are reviewed, focusing on data, metadata, web services and categories of users.

**Results:**

Though the web platforms are highly heterogeneous the review reveals that the latest advances in web technologies are underused. Information are rarely updated dynamically, metadata catalogues are absent, web services are more and more used, but rarely standardized, and websites are mainly dedicated to scientific communities, essentially researchers.

**Conclusion:**

Improvement of systems interoperability, through standardization, is an opportunity to be seized in order to allow real time information exchange and online multisource data analysis. To facilitate multidisciplinary/multiscale studies, the web of linked data and the semantic web innovations can be used in order to formalize the different view points of actors involved in the combat against malaria. By doing so, new malaria fighting strategies could take place, to tackle the bottlenecks listed in the United Nation Millennium Development Goals reports, but also specific issues highlighted by the World Health Organization such as malaria elimination in international borders.

**Electronic supplementary material:**

The online version of this article (10.1186/s12936-018-2270-0) contains supplementary material, which is available to authorized users.

## Background

Despite a steady fall in global malaria incidence rate, estimated to 18% from year 2010 to year 2016 [1], malaria remains a global public health preoccupation with an estimated 445,000 deaths globally in 2016 [1]. In 2000, the United Nations established the Millennium Development Goals (MDG) and defined the Goal 6: combat HIV/AIDS, malaria and other diseases; malaria was particularly referred to in Target 6C: have halted by 2015 and begun to reverse the incidence of malaria and other major diseases [2].

The end of the 15 years MDG agenda was definitely a milestone, a perfect time for the international community to balance achievements and set new objectives. Although the malaria MDG target 6C has been reached, malaria reasonably remained in the new United Nations schedule for the next 15 years called the Sustainable Development Goals (SDG) [3]. Malaria is part of the Goal 3: ensure healthy lives and promote well-being for all at all ages, and Target 3.3: by 2030, end the epidemics of AIDS, tuberculosis, malaria and neglected tropical diseases and combat hepatitis, water-borne diseases and other communicable diseases.

More than analysing achievements and providing objectives, the MDG 2015 report [2] identifies, for all millennium goals, bottlenecks that must be overcome. One of them is associated with the deficiency of data needed to monitor progress towards the MDGs: “*sustainable development demands a data revolution to improve the availability, quality, timeliness and disaggregation of data to support the implementation of the new development agenda”* [2]. If access to malaria diagnostics results is essential, other datasets are required in order to study the different processes involved in malaria epidemics, for example datasets coming from civil registration systems, among others. Furthermore, the need for a systematic evaluation of data quality is also highlighted. This information is to be recorded in the so-called metadata, data about data, in order to facilitate its exploitation but also to promote an objective and responsible use of it.

Another identified obstacle in combating malaria is the geographic and socioeconomic disparities of territories. Indeed, despite a global shared goal of eliminating malaria, researchers, stakeholders and policymakers are urged by the MDG 2015 report [2] to take into account territories heterogeneity, impelling them to work locally: “*Data at the local level proved extremely helpful*”. In this regard, national frontiers were identified in the literature [4–7] as critical locations for malaria—and any infectious disease—transmission; they represent as many bottlenecks for studying/preventing/intervening. A review of literature by Alimi [8] also asserts the importance of local studies for vector control in Latin America: “*Malaria elimination in this region will be difficult without locally tailored strategies for vector control*”. In this respect, previous studies [9–12] identified remote sensing images as a massive source of data for health monitoring and studying, at global scale, high spatial resolution, regularly updated and frequently inexpensive.

The World Health Organization (WHO) and the Global Malaria Programme (GMP), through the Global Technical Strategy for Malaria 2016–2030 [13], lists prevention and treatment strategies implemented to control and eliminate malaria. Mono-disciplinary approaches prevail and focus on vector control, chemoprevention and case management. They are mostly achieved by insecticide-treated mosquito nets (ITN), indoor residual spraying (IRS), intermittent preventive treatment and artemisinin-based combination therapy (ACT). However, recurrent associated difficulties, such as insecticide and artemisinin resistance [1], oblige the scientific community to increase its knowledge of malaria and more generally vector-borne diseases.

The MDG 2015 report [2] identifies the critical role every country has to play; one recommendation for them is to implement national open data policy and use international standards to share their information. In this scope, international cooperation is essential for standardization of data formats, acquisition procedures and infrastructures.

The increase of data volume and innovations in web technologies bring new opportunities but ask for theoretical studies in order to define how these resources will benefit malaria fighting [13]. They represent an occasion to tackle the bottlenecks previously listed. While creative people from technology areas daily introduce new tools and innovations, the persons in charge of combating malaria can clearly outline what is required and what already can be done. Particularly, malaria experts (researchers and operational actors) and computer science researchers working on data infrastructures could take the lead in developing new interdisciplinary methodologies and tools. Work has already started and, in the Geographic Information System (GIS) community, mapping tools are regularly used [14–16], online and/or off-line, for running heterogeneous data analysis or combining and communicating malaria risk information [17].

In this review, online platforms (websites, webpages) dealing with spatio-temporal information linked to malaria are explored in order to evaluate how web technologies are used and if they face the bottlenecks previously listed. In the second part, according to the previous results, propositions are discussed.

### Basic concepts

In this section some useful basic concepts used in the ongoing analysis are delineated.

**Fig. 1:**
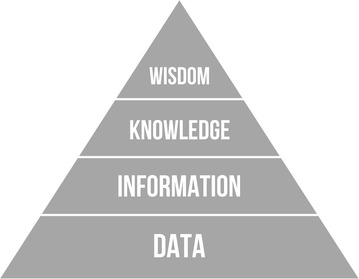
The DIKW pyramid

Data, information and indicator are interconnected concepts, the generally admitted difference between data and information is that whereas data is raw and has no sense for human beings, information derive from data and can be interpreted by humans. Contextualization is essential in order to turn data into information. An interesting tool for representing these two concepts is the DIKW—*Data*, *Information*, *Knowledge*, *Wisdom*—pyramid (Fig. 1) introduced by Ackoff in [18]. Even if taking a decision is more than applying finer-grained filters at each level of the pyramid, it symbolizes properly that quality of data—basement of the pyramid—is essential for obtaining quality information leading to an adequate action.

From a general perspective, an *indicator* is an information that mirrors a specific phenomenon. It is accepted by a community of users and could be associated to a domain of knowledge, like “ecological indicators”, “environmental indicators”, “economic indicators”, etc. In this paper, the focus is put on health indicators linked to malaria: “*A health indicator is a variable that can be measured directly to reflect the state of health of people within a community”* [19].

Indicators are commonly categorized into *objective* (e.g.: number of delivered ITN, budget of the ministry of health) and *subjective* (e.g.: happiness, health related quality of life), *analytic* (e.g.: child mortality, unemployment rate) and *synthetic* (e.g.: consumer price index, human development index) [20].

As noted in [20], the choice of an indicator is a compromise: whereas a *synthetic* one may be useful to reach a large community of users (including non-scientific people: citizens, stakeholders, etc.), its design (using multiple data sources and/or mathematical models) can make it inappropriate for studying complex processes; it can be difficult to validate, assess its bias, errors and the relative contribution of each factor to the studied phenomena, sometimes making *analytic* indicators more appropriate for this task [4].

*Metadata* consists in a resource description, or “data about the data”. In the web context, one metadata usage is to ease mediation: for computer and human being to research, access and process the data more easily. In this scope, metadata standards supply modelling languages, schemas and elements (controlled vocabulary) to record metadata. Metadata standards are diverse and numerous, the adoption of a standard depends on parameters such as its design intent but also its overall appropriateness by the community of users. Challenges and needs for metadata, in a dynamic digital information landscape, are described in [21] and a non-exhaustive list of standards are available in [22].

The use of Internet network revolutionized the way information was shared through web services; initially restricted to specialists—who were familiar with command lines—the development of dedicated webpages in the 90 s allowed for the general public to access remotely stored data. However, while more and more data are available online, there is a strong association between information and the website that make it available. In other words, if a user want to access information, in many cases he must use the user interface dedicated for distributing it, he has no alternative. Still, some technical solutions already exist, a “*web service is a software system designed to support interoperable machine*-*to*-*machine interaction over a network”* [23], it standardizes the online information exchange and allow for data access on third party clients, permitting new types of analyses, like simultaneous and remote multi-source data exploration. The capacity of systems to work together automatically (interoperability) is improved, and users are free to consult and download the data in different ways. Various standards already exist, some focus on geographical information, for example the Web Map Service (WMS) [24], that allows for georeferenced maps to be requested, or the Catalog Service for the Web (CSW) [25] that standardized the way catalogues of geospatial records are exposed on the Internet. A more popular standard, widely used for news synchronization, is the Rich Site Summary (RSS) [26] that allows users to be automatically alerted when news are published.

In this study, it will be useful to separate *server* and *client* side of web platforms. While the *server* is responsible, among other things, for implementing the technical solutions (web services) to broadcast the data and services, the *client* is the user interface, that provide online tools for researching, visualizing and analysing the data. In other words, the *client* is what the user sees on the webpages. Server-side and client-side are also known as back-end and front-end of a web platform.

## Methods

Websites where functionalities like consulting, visualizing and analysing spatio-temporal data linked to malaria are analysed. The study focuses on the “tool part” of the websites that is dedicated to data exploration (search/visualize/analyse). B*ing* and *google* search engines are initially used with the keywords “malaria *and* mapping”, “malaria *and* gis”, “malaria *and* online”. In order to include results that are not well indexed and do not show up from the first search method, websites are extracted indirectly from publications (articles, reports, posters) that are returned from the previous searches and from *google scholar*. Websites where users must register are not included. Completeness is not guaranteed, however these two research methods offer complementary results that are assumed to be representative of the global trend of how the web is used for searching, visualizing and analysing spatio-temporal data linked to malaria.

The criteria for analysing the websites are as follows:

### Data

#### Are multidisciplinary data available?

Data are listed and clustered into different fields of knowledge and websites are classified according to the level of multidisciplinarity they offer. The websites that give access to data originated from diverse fields of knowledge are separated from those that present multidisciplinary analysis or tools to encourage them, for example the possibility of simultaneous visualization of multiple data.

#### What spatial extents and units are used?

While malaria fighting is an undeniable global preoccupation that requires world level partnership, in the meantime it is also essential to conduct local studies. What kind of information is revealed by the available online datasets, are they suitable for running local studies?

Three expected levels of spatial extents are defined depending on the geographic coverage of a website. At *global* level, all regions are considered. At c*ontinental* level, all regions of a continent are represented. The r*egional* level is a sub-continental extent that goes from a locality (a district, a town, etc.) to a cluster of countries or regions (administrative, geographic, etc.).

**Fig. 2:**
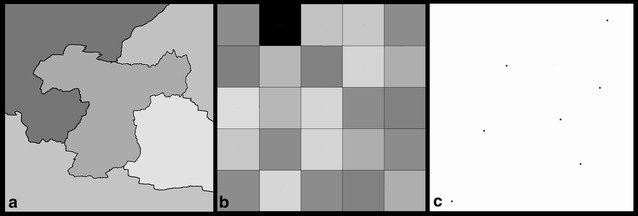
Representation of spatial units: thematic regions (**a**), regular grid (**b**) and isolated points (**c**)

Websites are classified depending on the spatial unit used for representing or analysing data. The two main categories of data used in Geographic Information Systems (GIS) are employed: vectors, when the territory is divided in *thematic regions* and/or when *isolated points, i.e.* spatially punctual information, are provided; and rasters, when the territory is observed through *regular grids*. Those categories are represented on Fig. 2.

#### Are data automatically/regularly updated?

While this information is neither systematically nor explicitly available, the way data are updated is a pointer to help identify what is the website intended for and what are the potential applications.

Websites are divided into two categories: the ones that provide a snapshot of a situation, presenting frozen information that never get updated, for example a web page dedicated to the publication of a specific study; and the ones that regularly and dynamically update their data, for example a web page that displays the air temperature in real time. These categories clearly present two different approaches of broadcasting information that we tag as *static* and *dynamic* respectively.

### Metadata

Is a metadata catalogue explicitly available and standardized? If metadata are present, do they follow international standards and what role do they have: data exploration (search), localization of data (access), data processing (analyse)? Finally, are data quality information explicitly available in metadata?

### Web services

*Client* and *server* sides of web platforms are separated in order to see how web-technologies are used for sharing, accessing and analysing information. The study focuses on two aspects:

#### Server-side

Is there an interoperable web service available for data access? i.e. is it possible to harvest data from another website or a local software?

#### Client-side

Are users allowed to upload their own data in order to cross them with those made available by the website?

Are tools available for analysing the data as *intuitive* functionalities? A functionality is tagged as *intuitive* when no computer science skills is needed to execute it. Non-expert users can intuitively implement it, the presence of a dedicated button to launch the service is a good indicator for it. A counterexample of an *intuitive* functionality is an interface where users have to use command lines to process the data.

#### Analysis tools are classified into three levels of functionalities

- *Basic:* customize the visualization picking up multiple information (e.g.: insecticide usage and insecticide resistance)
- *Advance:* extract a part of information or aggregate information (e.g.: define a region of interest and extract the corresponding data)
- *Complex:* combine indicators or run statistical analysis (e.g.: calculate the annual incidence rate from the annual incidence and the number of inhabitants or calculate a correlation coefficient between water level and malaria incidence).

### Categories of users

In order to classify the websites depending on *who* is likely to use them, four hypothetical (as the final users are rarely explicitly mentioned by the websites) *categories of users* and their respective expectations are defined: *health professionals*, *stakeholders/policy makers, researchers* and *civil society*.

**Fig. 3:**
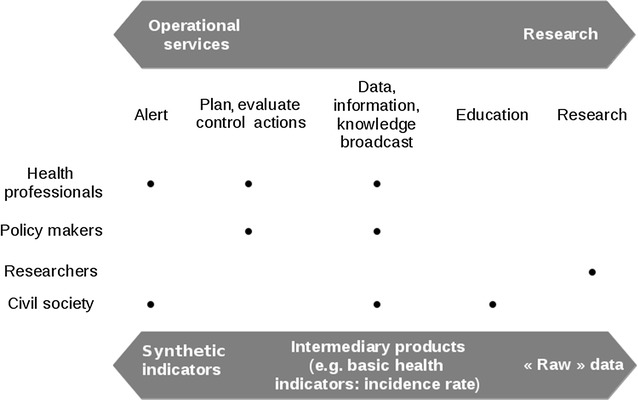
Categories of users, data types and services

While the *civil society* may expect information such as “where am I more vulnerable to malaria contamination” or “what strategy adopt to protect my 2 years old baby”, *health professionals* may be more sensitive to services such as “check the number of available treatments” or “consult the number of new cases in a specific district”. In the meantime, *stakeholders* and *policy makers* may look for indicators such as “an alert for an abnormal high epidemic-risk” or “an historical time series of malaria incidence”. In the case of *researchers*, an access to “raw qualified data”, “analysis results” and “tools to run analysis online” may be more adequate. This classification is represented in Fig. 3.

It is worth noting that the *categories of users* are not strictly separated and one person can switch from one category to another: for example a researcher can be interested in accessing the map of health centres during his/her holidays, or a policy maker may want to correlate the malaria incidence time series with the number of trained health agents. Thus, the term user’s *point of view* could have been used.

The *categories of users* are firstly determined according to the type data: are indicators objective or subjective, analytic or synthetic? Secondly, the way an information is introduced is analysed: are users guided for interpreting it? Is there an information synthesis systematically displayed for introducing it?

## Results

Eleven websites were found, they are listed in the references [27–37] and in Table 1: they are labelled from *a* to *k*. Some websites offer multiple searching, visualizing and/or analysing interfaces; as they are technologically close, we refer to them jointly.

**Table 1:** Names, URLs and labels of the reviewed websites

Website name	URL	Label
Malaria Atlas Project	http://www.map.ox.ac.uk/explorer/ http://www.map.ox.ac.uk/country-profiles/	a
IRI Climate and Malaria in Africa	http://iridl.ldeo.columbia.edu/maproom/Health/Regional/Africa/Malaria/	b
IR Mapper	http://www.irmapper.com	c
Drug Resistance Maps	http://www.drugresistancemaps.org	d
HealthMap	http://www.healthmap.org	e
VectorMap	http://vectormap.nhm.ku.edu/vectormap/	f
Worldwide Antimalarial Resistance Network	http://www.wwarn.org/explorer/app/ http://www.wwarn.org/molecular/surveyor/k13/ http://www.wwarn.org/dhfr-dhps-surveyor/ http://www.wwarn.org/molecular/surveyor/ http://www.wwarn.org/aqsurveyor/	g
VectorBase	http://www.vectorbase.org/popbio/map/ http://www.vectorbase.org/rest/	h
MalariaGen	http://www.malariagen.net/apps/pf/ http://www.malariagen.net/apps/pvgv/ http://www.malariagen.net/apps/pf3k/ http://www.malariagen.net/apps/ag1000g/	i
MalariaThreats	http://apps.who.int/malaria/maps/threats/	j
StatCompiler	http://www.statcompiler.com	k

**Fig. 4:**
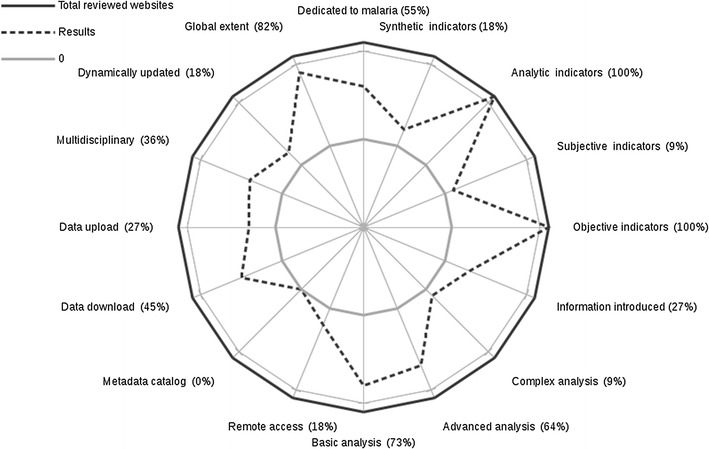
Results preview of the reviewed websites

The results of this review are synthesized partly in Fig. 4 and completely represented in the additional table file; they are detailed hereafter.

### Data

From the eleven reviewed websites, six (a, b, d, g, i, j) deal exclusively with malaria, the remaining ones also consider other diseases.

#### Multidisciplinarity

The eleven websites offer a large amount of indicators, the main ones being listed and clustered in Table 2. The term *field of knowledge* encompasses the terms *scientific fields* and *scientific/operational issues*.

**Table 2:** Fields of knowledge and linked indicators

Field of knowledge	Indicators
Drug resistance	Halotype, mutation type, molecular type resistance, drug type, marker, d28 efficacy
Insecticide resistance	Insecticide class, resistance mechanism
Intervention	Vector control strategies (larval control, IRS), treatment (ACT, intermittent preventive treatment), personal protection (ITN)
Entomology	Dvs occurrence, vector occurrence, vector bionomics, reproductive number, temperature suitability, entomological inoculation rate, mosquitoes presence, vectorial capacity, phenotype, vector species
Epidemiology	In/out patient, death, population at risk, incidence, prevalence, pf/pv parasite rates, g6pd deficiency, sickle cell, duffy negativity, haemoglobin c, clinical burden, population at risk, endemicity, transmission limit, seasonal climatic suitability for malaria transmission, malaria seasonality, relapse incidence, fever cause
Malaria facing strategies/policies	Free treatment, collaboration public/private sector, mandatory case reporting, new cases/relapse distinction, active/passive surveillance, case detection techniques (microscopy, rapid diagnostic test)
Geography	Landcover, district frontiers, administrative boundaries, schools, hospitals, health services
Demography	Population statistics
Environment	Precipitation, temperature, vegetation, land surface temperature, meteorology/climatology, hydrology
News	Google news, ProMED Mail, WHO, Baidu News, SOSO Info
Sociology	Education, urban/rural, wealth index, travel time to cities

Taken as a whole, the use of information originating from multiple fields of knowledge is clear. Still, not every website offers the same level of heterogeneity: from the eleven websites, five (c, d, e g, i) work with information that come from a unique field of knowledge; the others present information that come from three or more fields of knowledge. However, two of them (h, j) only allow for monodisciplinary analysis or visualization of data; the four remaining ones (a, b, f, k) effectively allow for multidisciplinary approaches to be carried out, mainly by providing visualization tools for combining data or by exposing results from previous multidisciplinary studies.

Thus, four out of eleven reviewed websites (36%) are tagged *multidisciplinary* and the others *monodisciplinary*. Some fields of knowledge are underrepresented: *news* and *sociology* only appear in one (e) and two (a, k) websites, respectively.

#### Spatial study extent

From the eleven websites, a majority of nine (82%) gives access to information at *global* level, i.e. they provide data for all territories. The two remaining websites, *IRI Climate and Malaria in Africa* (b) [38] and *Drug Resistance Maps* (d) exclusively focus on a *continent* (Africa).

#### Spatial study unit(s)

##### Thematic regions

The *StatCompiler* website (k) exclusively presents information at national and/or subnational levels, with, for each considered parameter, one value for the entire national country and/or for each subnational administrative region.

##### Regular grids

In *the IRI Climate and Malaria in Africa* Website (b), data is represented quasi continually on the entire studied territory with a constant spatial resolution. Data mainly originate from remote sensing images, the resulting information has a typical 1–5 km spatial resolution, reaching 250 m in the case of the vegetation characterization extracted from *modis* sensor.

##### Isolated points

Another way of representing information is to refer to it using a unique geographical point. This is the case of seven (64%) of the reviewed websites (c, d, e, g, h, i, j). If the geographical point is occasionally directly linked to the data, for example the location of an in situ sensor, it frequently refers to the place where the information was processed, for example the location of the laboratory that analysis the data, not the location of the field camp where the data was acquired.

The last two websites (18%) (a, f) give access to a large heterogeneous panel of data using different spatial units. They both allow for visualizing *regular grid* and *isolated points* data jointly on a map. In the *Malaria Atlas Project* website (a) [39, 40] it is also possible to display *thematic regions* (country) information.

Then, the majority of websites uses the *isolated points* category of spatial unit. As already said, in this category, the spatial information is not always directly related to the data acquisition. However, four websites take advantage of this spatial information by: aggregating dynamically information (number of studies but also their results) depending on the zoom level in the *VectorBase* website (h) [41], giving quick access to a list of results by country in the *Worldwide Antimalarial Resistance Network* website (g) [42], or providing a shortcut to the number of studies at country level in *MalariaGen* (i) and *MalariaThreats* (j) websites.

#### Data update

From the eleven evaluated websites, two are classified *dynamic*: the *IRI Climate and Malaria in Africa* (b) and *HealthMap* (e) [43] websites. They automatically update their information by directly requesting the distant data sources: as soon as the data is released by the data producer they make it available. Even if the remaining websites present up to date results, manual procedures are used to upload new information: they are labelled *static*.

### Metadata catalogue and metadata standards

From the eleven evaluated websites, none of them offers functionality centred on a standardized metadata catalogue.

### Web services

#### Server side

From the eleven websites, two of them (b, h) provide standardized services allowing for data access independently of the use of their own client. While *j* distribute the data via a REpresentational State Transfer (REST) endpoint, *b* also gives access to data via a WMS web service. The large majority (82%) of the reviewed websites only allow for data access through their dedicated web pages.

#### Client side

It is possible to import and visualize remotely stored data in two websites (a, c). Strategies to upload data are the same: a template can be downloaded and users manually format their local data before uploading them. However, goals are different: while the *IR Mapper* website (c) [44] allows for visualizing the uploaded information, the *Malaria Atlas Project* website (j) does not. It uses the uploaded locations (region/points) as a parameter for extracting information already contained in the website. The other nine websites (82%) do not allow for external data to be imported, resulting in analysis possibilities limited to their respective data.

The eleven websites use a dynamic map and/or graph that allows interacting (zoom in and out mainly) and requesting for information to be displayed by clicking on the point of interest. In eight websites (73%) (a, c, e, f, g, h, i, k), as an *intuitive* functionality, users can select data of interest and display them on a same map/graph. This functionality corresponds to the first level of analysis (*basic*) we detailed in the methodology part.

The second level of analysis (*advance*: extract or aggregate information) is available as an intuitive functionality in seven websites (64%). The *Malaria Atlas Project* website (a) allows to define a region (or points) of interest by uploading a file or selecting a predefined administrative region (country and subnational region) in order to extract information. The *IRI Climate and Malaria in Africa* website (b) allows to define a region of interest and download the corresponding information. The *HealthMap* website (e) allows to select a region of interest (town, subnational region, country, continent) and visualize the past year evolution of the corresponding number of disease alerts. The *Worldwide Antimalarial Resistance Network* website (g) allows to compare two study results by visualizing them jointly. The *VectorBase* website (h) allows for aggregating data depending on the zoom level and to visualize study results jointly on a graph. It is also possible to download the selected information. The *MalariaGen* website (i) [45, 46] allows to play with genome information through a “genome browser”. Finally, the *StatCompiler* website (k) allows to explore and personalize graphs by the way of a dynamic menu.

The third level of analysis (*complex*: combine information) is only available as an intuitive functionality in the *MalariaGen* (i) website: an ergonomic interface allows to build an advanced processing chain in order to process genome information. However, as a *non*-*intuitive* functionality, *b* also allows to run *complex* analysis through a dedicated programming language named *Ingrid.*

### Category of users

#### Indicator types

Even if it mainly provides objective information, the *StatCompiler* website (k) also deals with subjective data: “social and economic well-being”. The remaining ten websites only provide objective data. A majority of nine websites (82%) (a, c, d, e, f, g, h, i, j) deals only with analytic indicators, the remaining ones (b, k) deal with both analytic and synthetic indicators, however, they are mainly analytics.

#### Introduction of information

Four websites (36%) clearly make efforts for democratizing access to scientific information by giving essential keys for interpreting the displayed results; i.e. it is possible to visualize jointly the results and a short text explaining them. This is the case of *a*, *b*, *j* and *k* that the *civil society* category of user may consult. The *HealthMap* website (e) may also interest this category of users as it centralizes news about health alerts.

The category of *health professionals* may be interested in websites that provide information that help to evaluate a health situation, plan and generate alerts; this is the case of *b*, *e* and *k* that relate news, health statistics and warning indicators.

*Stakeholders/Policy makers* may also find interesting those websites and they are likely to use *Malaria Atlas Project* (a) and *Malaria Threats Map* (j) websites that pre-process output products, like ready to use maps and reports that can be downloaded as image files.

These data format (png, jpeg or pdf) do not allow for *researchers* to process them, they may be more interested in downloading raw data as they can do in *a*, *b*, *g*, *h* and *k* websites. Four websites (c, d, f, g) gather previous studies that focus on a same topic in an implicit goal of exhaustiveness. Users can explore the datasets by choosing a study result, for example: “confirmed resistance”, “possible resistance”, etc. Those websites do not immediately present indicators, but rather the study that produced them. According to our classification, they are clearly dedicated to the category of *researchers,* it is unlikely for another category of users to have interest in those resources. The last website that may interest the *researchers* category is the *MalariaGen* website (i) that provides a tool for running *complex* analysis.

In short and to our subjective appreciation (due to the fact that none of the websites explicitly mentions targeted categories of users): *researchers* may find useful nine websites (82%), *stakeholder/policy makers* 5 (45%), *health professionals* 3 (27%) and the *civil society* 5 (45%). Those results are summarized in Additional file 1.

## Discussion

### Data

This review reflects a minority usage of the web for communicating or encouraging multidisciplinary studies: four out of eleven reviewed websites (36%) actually have a multidisciplinary approach. It also reveals that some disciplines are underrepresented (sociology, news) or even absent (population dynamics for example). Working with multidisciplinary data implicates being able to work with different spatial extents and units, parameters that are directly linked to data availability and collecting procedures, study objectives, storage and computing constraints. To facilitate multidisciplinary studies, adequate approaches and tools for representing, modelling and co-analysing the spatially heterogeneous datasets must be developed. Mathematical models for interpolating low spatial resolution or punctual data combined with high spatial resolution remote sensing images is already used in the *Malaria Atlas Project* website (a), but upstream approaches may be more powerful and facilitate multidisciplinary and/or multiscale analysis. For example, how to automatically evaluate what spatial extent and unit are adequate to address a specific question? While a 25 km^2^ grid can be considered satisfactory for climate scientists, it is still too coarse for mosquitoes breeding habitats mapping and vector control. It behooves to every knowledge area to determine the spatial characteristics they believe suitable for their study. In the context of the web of data, and particularly the semantic web, standardized languages, schemas and tools already provide a framework for knowledge modelling, sharing and automatic inferring. Particularly, for recording the different viewpoints in relation to space scales and units, the OGC standard GeoSPARQL [47] and the stRDF/stSPARQL initiative [48] provide vocabularies and query languages that can be used. Inherently, easy sharing and automatic inferring would be possible, facilitating multidisciplinary and/or multiscale analysis. Crossborder multilateral studies, characterized by different viewpoints in relation to a territory (a simple example is the one of administrative divisions) could also be facilitated.

It turned out to be challenging to retrieve what is the update frequency of a website and if a dataset is up-to-date. When data is systematically and routinely produced, the recommended update frequency corresponds to the time resolution of the data. It may vary from day to year depending on the website: *VectorMap* (f) [49] displays the monthly malaria incidence rate and *StatCompiler* (k) the annual malaria incidence rate. The first one is to be updated once a month, the other one every year. Compared to automatic procedures, manual procedures inevitably lead more easily to “outdated” information, even more when data come from multiple sources, are heterogeneous and need preprocessing steps in order to make them comparable. The second main type of data available is the collection of punctual non-recurrent studies and results, for example the mapping of a city’s districts, a Chloroquine efficacy clinical trial or a news. The accurate moment to update information instinctively matches the date a new result is made available. It turns out to be trickier for that type of data to evaluate what are the adequate criteria to state if a data is “outdated”; we can recommend the use of a “flag” for the data producer to indicate this notion, it could be part of the quality information available in the metadata, as completeness, representativity and reliability.

### Metadata

Making scientific data and research more visible and accessible for everyone is in the scope of the “Open science” dynamic. Beyond making the data available on the Internet is also the need for providing keys for self-appropriation of information by every community of users. Metadata is such a tool, by systematically describing the data in a dedicated framework, ideally in a standardized way, the user is able to access key information such as data origin or data processing steps. This review outlines the underutilization of this tool: no website uses a metadata catalogue and it is not possible to access metadata files. Yet, metadata exist as the websites need them for researching, locating and providing information about the data; but they are not visible for the user. In order to promote infrastructures interoperability, in addition to using standardized metadata catalogue, it would be usefull to use systematically a small set of vocabulary terms, generic and easy to use, for describing the datasets: the Dublin Core Metadata Element Set (DCMES) [50]. Other standards have thematic purposes and are richer than the previous one, for example the ISO19115 standard [51] that focuses on the description of geographic information.

A need for systematic data quality assessment and dissemination was also noted. While scientific works systematically involve result quality estimation, this information should be made accessible jointly with the data. None of the eleven websites do so systematically.

### Web services

This review reveals that the large majority of web platforms provides limited web services; they do not allow for third party solutions to directly access their data and the large majority of websites does not provide tools for running complex analysis online. For example, the upload of data originated from other sources is only possible in two websites. However, we noted that websites are regularly updated in order to provide new tools, mainly for running analysis, more and more web platforms implement innovative technical solutions that allow for every user to take advantage of technology improvements, such as Internet network speed or processing capacity. In the same way as Google Earth Engine (GEE) online platform does [52], it is possible to concentrate on a same website both a free and easy access to large heterogeneous datasets and a library for processing data on a cluster of high performance computers.

Still, the two websites that allow running *complex* analysis (b, i) use non-standardized technologies (programming languages, communication protocols) that makes any processing chain development specific to the platform. If the platform is down, if the company bankrupts or decide to change its terms of use, there could be no chance for reproducing the analysis. Some users still hesitate to enjoy these technologies for those reasons; however the use, or development and publication, of open solutions can guarantee reproducibility and sustainability. The *MalariaGen* website (i) actually provides the source code of the web application *Panoptes* [46, 53], permitting a local installation of the software. Concerning web services, two main approaches are used to ensure interoperability: the OGC Web Processing Service (WPS) that standardizes “*how clients can request the execution of a process, and how the output from the process is handled.”* defining *“an interface that facilitates the publishing of geospatial processes and clients’ discovery of and binding to those processes.”* [54], and the REST architecture style [55], already used by *b* and *h*, that specifies constraints to adopt for ensuring services to work best on the Web.

### Categories of users

In the *Basic concepts* part of this review, the usefulness and efficiency of synthetic indicators for communicating complex information to a large diversity of users were highlighted. However, this type of indicators is hardly used by the reviewed websites, the result is the same for subjective indicators that are almost never used.

Analysing the way information is introduced to users, we identified five websites as useful for the *stakeholder/policy makers* category. However, only one of them (b) provides a synthetic indicator intended to alert in real time that a high epidemic risk is pending by displaying the results of a vectorial capacity model [56]. Still, interesting researches projects exist and could lead to operational methods and tools. For example: information extraction from text and particularly from newswires [57], the implemented solutions allow for worldwide epidemic surveillance, they represent an interesting data source to be integrated to multidisciplinary studies; in Madagascar, an automated malaria outbreak detection system [58] was developed, using statistical detection methods to analyse data coming from a network of primary health care centres, weather data and malaria intervention statistics. Mobile phone network (Short Message Service—SMS) is used for communicating disease notifications and analysis feedbacks, including alerts.

Five websites were identified as useful for the *civil society* category. The principal criterion is to look if information are vulgarized. Such efforts are made in heterogeneous ways and users rapidly need a scientific background to not misunderstand information. Outside the reviewed websites, an interesting one was found, the international network of health information (Rede Interagencial de Informações para a Saúde—RIPSA) website [59] that provides a dedicated web page for describing each indicator. An eloquent example is the one of the Annual Parasite Index (API) [60] that describes the indicator, from the mathematical formula to the interpretations and limitations. This kind of tool is useful for every category of users but could be systematically used when focusing on the *civil society* category.

Only three websites may interest the defined *health worker* user category. Practical working tools were looked for, for example to delimit epidemiological districts in a town, or record new malaria cases. However, the literature relates that some initiatives actually exist and focus on this category of users, with promising results: in Thailand, a dedicated project [61] was carried out to implement a platform permitting online/offline data entering for malaria staff; the article outline the improvement of the quality of Thailand’s malaria system. In Pakistan, an online system was implemented [62] which permits to acquire, store and analyse patients’ data in real time (not only for malaria), facilitating health workers jobs but also opening new analysis opportunities for researchers and possibilities for emitting real time epidemiological alerts. Malaria reporting quality was also evaluated in Brazil [63, 64] thanks to the Malaria Epidemiological Surveillance Information System (Sivep-Malaria). Those web platforms were not reviewed as a login/password was necessary to access them. The “Open science” dynamic, that aims at turning scientific data and researches more visible and accessible for everyone rises specific issues concerning data privacy in the case of health data. In order to build a Health Web Observatory (HWO), Gloria [65] highlights the need for “an additional layer of legal and ethical considerations” that “requires an understanding of multiple levels of complexity that include individual, social, and legal factors”. Limited access to some data fields seems inevitable to guarantee data privacy. This is another application of metadata catalogue: an authentication step could be necessary to visualize some information (for example the number of malaria cases), but the associated metadata records would remain available for everyone.

The nine websites classified as useful for the *researchers* category basically provide at least one of these functionalities: give access to raw data, catalogue previous studies on a precise subject and provide analysing tools. This category of users seems to take more advantage of web technologies than the others, one explication is that the different websites mainly originate from this community.

## Conclusion

In this review a reading matrix was defined in order to run a comparative study of web platforms and assess their added values in the scope of the malaria elimination objective and in regard to technological advances. A section was dedicated to discuss the different user’s expectations regarding the websites. Objective criteria were introduced to categorize different types of services and subjective criteria to link them to four hypothetical *categories of users*.

Individual recommendations are not relevant in the context of rapid evolution of websites (creation, addition of new functionalities), however, eleven heterogeneous and representative (in respect to functionalities, technologies and involved institutions) web platforms dealing with spatio-temporal malaria scientific information were reviewed and general recommendations made for guiding future web platforms developments.

The use of web technologies and international open standards and normalization for cataloging, analysing and broadcasting data can help to draw a new model for studying and combating malaria and other vector-borne diseases. A need for a new type of technology usage was identified; one objective is to involve a broader spectrum of users by providing a greater and easier access to stimulating “real-time” information, in accordance with the MDG 2015 report [2]. *Dynamic* web platforms are appropriate for this task: automatically updated, persistently and at user-request: the data is automatically updated, at every consultation. However, the majority of *static* websites reviewed, that provide information generally linked to time limited research projects, clearly turned out to be efficient medias for communicating information, but they must be paired to scientific publications and vulgarization tools for allowing a large dissemination. In this regard, the use of standardized metadata and metadata catalogues would permit an easier dataset exploration, but also provide a framework for registering essential information such as data quality (including an “obsolescence” parameter) and information allowing for reproducibility. The use of metadata also turned-out to be a trick for allowing critical data (in respect to privacy) research and localization without allowing its visualization.

Another objective is to facilitate multidisciplinary studies. The use of standardized web services for accessing heterogeneous datasets brings multidisciplinary analysis opportunities. However, new challenges are to be faced to reach this objective, developing web platforms for analysing data online seems relevant, for every user to be able to access high performance infrastructures and processing chains, without having to download big volume of distributed data. However, while some solutions already exist, even though they take advantage of open Internet standards and protocols, they introduce non-standardized solutions. Finally, in order to facilitate multidisciplinary studies, research works must be led to facilitate knowledge modelling; the use of standards and tools from the semantic web is a promising possibility, for example in respect to information representation units.

## Additional file

**Additional file 1.** Results preview of the reviewed websites.

## Abbreviations

ACT: artemisinin-based combination therapy
API: Annual Parasite Index
CSW: Catalog Service for the Web
DCMES: Dublin Core Metadata Element Set
GEE: Google Earth Engine
GIS: Geographic Information Systems
GMP: Global Malaria Programme
HTTP: HyperText Transfer Protocol
HWO: Health Web Observatory
IRI: International Research Institute for climate and society
IRS: indoor residual spraying
ITN: insecticide-treated mosquito nets
MDG: Millennium Development Goals
REST: REpresentational State Transfer
RIPSA: International network of health information (Rede Interagencial de Informações para a Saúde)
RSS: Rich Site Summary
SDG: Sustainable Development Goals
SMS: Short Message Service
USAID: United States Agency for International Development
WHO: World Health Organization
WMS: Web Map Service
WMTS: Web Map Tile Service
WPS: Web Processing Service
